# Cardiovascular and Renal Risk Factors and Complications Associated With COVID-19

**DOI:** 10.1016/j.cjco.2021.05.020

**Published:** 2021-06-16

**Authors:** Rhian M. Touyz, Marcus O.E. Boyd, Tomasz Guzik, Sandosh Padmanabhan, Linsay McCallum, Christian Delles, Patrick B. Mark, John R. Petrie, Francisco Rios, Augusto C. Montezano, Robert Sykes, Colin Berry

**Affiliations:** Institute of Cardiovascular and Medical Sciences, British Heart Foundation, Glasgow Cardiovascular Research Centre, University of Glasgow, Glasgow, United Kingdom

## Abstract

The current COVID-19 pandemic, caused by the severe acute respiratory syndrome-coronavirus-2 (SARS-CoV-2) virus, represents the largest medical challenge in decades. It has exposed unexpected cardiovascular vulnerabilities at all stages of the disease (pre-infection, acute phase, and subsequent chronic phase). The major cardiometabolic drivers identified as having epidemiologic and mechanistic associations with COVID-19 are abnormal adiposity, dysglycemia, dyslipidemia, and hypertension. Hypertension is of particular interest, because components of the renin–angiotensin system (RAS), which are critically involved in the pathophysiology of hypertension, are also implicated in COVID-19. Specifically, angiotensin-converting enzyme-2 (ACE2), a multifunctional protein of the RAS, which is part of the protective axis of the RAS, is also the receptor through which SARS-CoV-2 enters host cells, causing viral infection. Cardiovascular and cardiometabolic comorbidities not only predispose people to COVID-19, but also are complications of SARS-CoV-2 infection. In addition, increasing evidence indicates that acute kidney injury is common in COVID-19, occurs early and in temporal association with respiratory failure, and is associated with poor prognosis, especially in the presence of cardiovascular risk factors. Here, we discuss cardiovascular and kidney disease in the context of COVID-19 and provide recent advances on putative pathophysiological mechanisms linking cardiovascular disease and COVID-19, focusing on the RAS and ACE2, as well as the immune system and inflammation. We provide up-to-date information on the relationships among hypertension, diabetes, and COVID-19 and emphasize the major cardiovascular diseases associated with COVID-19. We also briefly discuss emerging cardiovascular complications associated with long COVID-19, notably postural tachycardia syndrome (POTS).

## Résumé

La pandémie actuelle de COVID-19 causée par le coronavirus du syndrome respiratoire aigu sévère 2 (SRAS-CoV-2) est le plus grand enjeu médical des dernières décennies. Elle a mis en évidence des vulnérabilités cardiovasculaires imprévues à tous les stades de la COVID-19 (avant l'infection, pendant la phase aiguë et pendant la phase chronique subséquente). Les principaux facteurs cardiométaboliques dont les associations épidémiologiques et mécanistiques avec la COVID-19 ont été avérées comprennent l'adiposité anormale, la dysglycémie, la dyslipidémie et l'hypertension. L'hypertension suscite un intérêt particulier, car certaines composantes du système rénine-angiotensine (SRA), dont le rôle est crucial dans la physiopathologie de l'hypertension, sont également en cause dans la COVID-19. Plus précisément, l'enzyme de conversion de l'angiotensine 2 (ECA2), une protéine multifonctionnelle du SRA faisant partie de l'axe protecteur du SRA, est également le récepteur permettant au virus SRAS-CoV-2 d'entrer dans les cellules hôtes et de provoquer une infection virale. Les affections cardiovasculaires et cardiométaboliques concomitantes ne font pas que prédisposer les personnes qui en sont atteintes à la COVID-19, elles constituent également des complications de l'infection à SRAS-CoV-2. En outre, de plus en plus de données probantes indiquent que l'atteinte rénale aiguë est fréquente en cas de COVID-19, qu'elle survient tôt et fait l'objet d'une association temporelle avec l'insuffisance respiratoire, et qu'elle est associée à un pronostic sombre, notamment en présence de facteurs de risque cardiovasculaires. Nous discutons ici des maladies cardiovasculaires et rénales dans le contexte de la COVID-19, et présentons les progrès récents sur les mécanismes physiopathologiques en cause dans le lien entre les maladies cardiovasculaires et la COVID-19 en nous attardant sur le SRA et l'ECA2, ainsi que sur le système immunitaire et l'inflammation. Nous présentons de l'information à jour sur les liens entre l'hypertension, le diabète et la COVID-19, et soulignons les principales maladies cardiovasculaires associées à la COVID-19. Nous analysons également brièvement les complications cardiovasculaires émergentes associées à la COVID-19 de longue durée, notamment le syndrome de tachycardie orthostatique posturale (STOP).

The global ramifications of COVID-19, caused by severe acute respiratory syndrome-coronavirus-2 (SARS-CoV-2), have been far-reaching, impacting the health of millions, straining national healthcare systems worldwide, and weakening global economic stability.^1^^,^^2^ Although presenting clinically as a respiratory infection, COVID-19 increasingly is being regarded as a systemic disease not solely restricted to the respiratory system. Patients hospitalized for COVID-19 have been found to also have increased rates of septic shock, acute kidney injury, rhabdomyolysis, and disseminated intravascular coagulation (DIC).^3^ Moreover, in addition to the lungs, COVID-19 has been found to cause dysfunction of multiple organs, affecting the heart, kidneys, and liver,^4^^,^^5^ and SARS-CoV-2 has also been postulated to invade the central nervous system, as do other coronaviruses, although conclusive data are lacking.^6^ Emerging evidence clearly indicates complex interactions between COVID-19 and the cardiovascular system, with poorer outcomes for those with underlying comorbidities, and the possibility of direct and long-lasting cardiovascular damage.^7^^,^^8^

**Figure 1:**
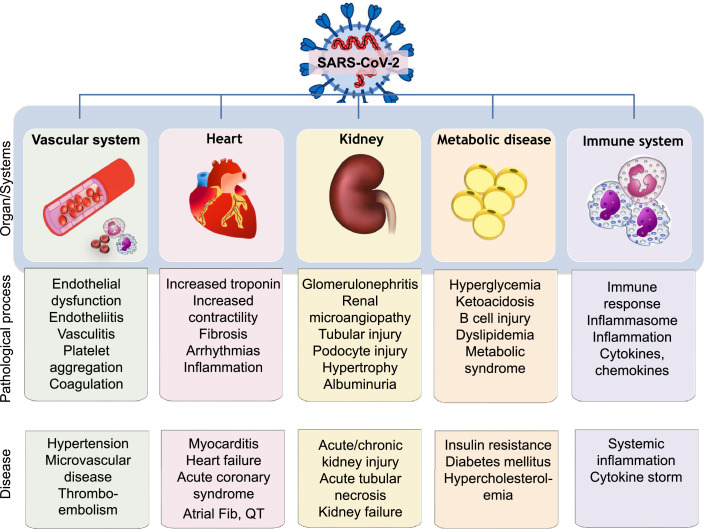
Diagram demonstrating components of the renin–angiotensin system and the multifunctional role of angiotensin-converting enzyme 2 (ACE2). ACE2 acts primarily as an enzyme to generate angiotensin (Ang)-(1-9) and Ang-(1-7) from Ang I and Ang II, respectively. ACE2 is also the receptor for severe acute respiratory syndrome coronavirus 2 (SARS-CoV-2) that causes coronavirus disease 2 (COVID-2). Fib, fibrillation.

Since the identification of SARS-CoV-2 in humans, a multitude of cardiovascular complications, including myocardial injury, heart failure, arryhthmias, and thromboembolic disease, as well as kidney disease, have been reported, with approximately 1 in 4 patients affected^9^^,^^10^ (Fig. 1). Physiological stress due to hypoxia, hypotension and tachycardia; provocation of acute coronary syndromes or arrhythmia; direct viral infiltration; and the effects of systemic inflammation and coagulopathies are implicated. Additionally, preexisting coronary artery disease and cardiovascular risk factors such as diabetes, obesity, chronic kidney disease, and hypertension are associated with increased risk of severe COVID-19 infection and mortality.^11^^,^^12^ These associations have been linked to the important role of angiotensin-converting enzyme 2 (ACE2), a component of the renin–angiotensin system (RAS), and the receptor through which SARS-CoV-2 mediates infection.^13^, ^14^, ^15^, ^16^ The connection between SARS-CoV2 infection and cardiovascular disease is not new, as other viruses in the Coronaviridae family, including SARS-CoV and Middle East respiratory syndrome (MERS-CoV), have long been known to be associated with myocarditis and heart disease, possibly through ACE2 tropism.^17^, ^18^, ^19^, ^20^, ^21^

This review provides an overview of cardiovascular, cardiometabolic, and kidney diseases as risks associated with COVID-19 and as complications and long-term sequelae of SARS-CoV-2 infection. We also highlight the importance of ACE2, a component of the RAS, and inflammation, as key factors in COVID-19–related cardiovascular disease.

## ACE2 as the SARS-CoV-2 Receptor, and Cardiovascular Implications

ACE2 is a carboxypeptidase that exists in both membrane-bound and soluble forms. The majority of the protein that comprises the N-terminal domain, including the catalytic site, of membrane-bound ACE2 is oriented extracellularly, with a transmembrane domain anchoring it to the cell membrane. The soluble form of ACE2 is cleaved and shed as the N-terminal ectodomain and is typically found in the circulation at low concentrations, although it may increase under pathologic conditions.^22^, ^23^, ^24^ The primary physiological role of ACE2 in the RAS is its catalytic function to produce angiotensin-(1-9; Ang-(1-9)) and angiotensin-(1-7; Ang-(1-7)) from Ang I and Ang II, respectively.^25^ The major product of ACE2 activity is Ang-(1-7), which binds to the Mas receptor, inducing vasodilation and antiproliferative, anti-inflammatory, antifibrotic, anti-thrombotic, and anti-arrhythmogenic effects that provide cardiovascular protection.^26^, ^27^, ^28^ The sequence of events culminating in Mas receptor activation is referred to as the ACE2-Ang-(1-7)-Mas receptor axis and represents the protective side of the RAS.^28^, ^29^, ^30^

**Figure 2:**
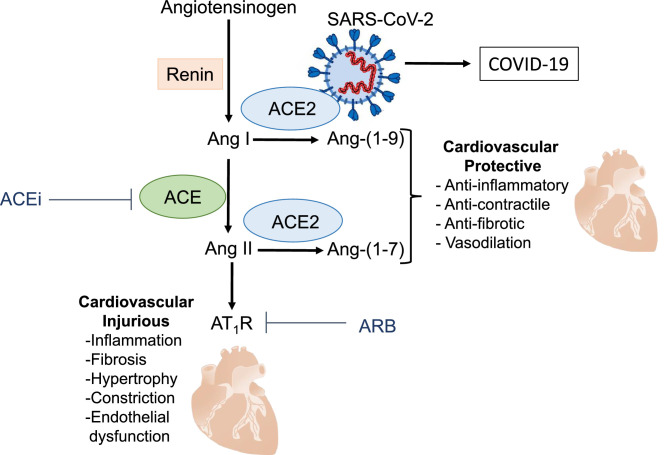
COVID-19 and cardiovascular and cardiometabolic disease. COVID-19 is a respiratory disease, but it is also associated with vascular dysfunction, coagulopathies, myocardial injury, metabolic disturbances, kidney injury, and systemic inflammation. These processes contribute to widespread cardiovascular and metabolic pathologies, including hypertension, heart disease, thromboembolic disease, kidney disease, and cytokine storm. ACE2, angiotensin-converting enzyme 2; ACEi, ACE inhibitor; Ang, angiotensin; ARB, angiotensin receptor blocker; AT_1_R, angiotensin II type 1 receptor; COVID-19, coronavirus disease 2019; SARS-CoV-2, severe acute respiratory syndrome coronavirus 2.

COVID-19 is attributed to SARS-CoV2, which uses ACE2 as its host cell entry receptor^31^, ^32^, ^33^ (Fig. 2). ACE2 is widely expressed and is found in the heart, kidneys, testes, type 2 alveolar epithelial cells of the lung, enterocytes of the small intestine, and endothelial and vascular smooth muscle cells of arteries, veins, and lymphatics.^34^^,^^35^ The tissue distribution of host entry receptors is believed to coincide in a general sense with viral tropisms, and theoretically, SARS-CoV-2 may be able to enter and infect any cell or tissue that expresses ACE2.^36^^,^^37^ However, this notion has been challenged, as ACE2-expressing human intestinal cell lines failed to be infected by SARS-CoV.^38^ Similar findings have been reported for SARS-CoV-2, for which there is a lack of evidence of ACE2 expression and replicative infection in human endothelial cells.^39^ Expression of ACE2 alone may not be sufficient for viral infection, and other factors, such as transmembrane protease serine 2 (TMPRSS2), cathepsin, and other proteases and binding proteins may be essential.^40^^,^^41^

Although the primary target of SARS-CoV-2 is the respiratory epithelium, there is some evidence that the virus, via ACE2, directly invades cardiomyocytes of the heart, causing viral myocarditis.^42^^,^^43^ Supporting evidence in experimental models showed that pulmonary infection with SARS-CoV resulted in an ACE2-dependent myocardial infection, with subsequent decreased ACE2 expression.^44^

Beyond its role as the SARS-CoV-2 entry receptor, ACE2 might play an indirect role in the pathophysiology of COVID-19 by influencing the inflammatory response. It may be possible that SARS-CoV-2 binding to ACE2 causes downregulation of ACE2, with reduced cleavage of Ang II, to generate anti-inflammatory Ang-(1-7). This process would upregulate the detrimental ACE-Ang II-angiotensin II type 1 receptor (AT_1_R) axis and downregulate the protective ACE2-Ang-(1-7) axis.^30^^,^^45^ Although plausible, this possibility has yet to be unambiguously demonstrated and proven. However, considering the pathophysiological role of the ACE2-Ang-(1-7)-MasR axis in diabetes mellitus, hypertension, atherosclerosis, heart disease, and kidney disease, underlying cardiovascular pathologies may be acutely exacerbated, or potentially chronically worsened, by SARS-CoV-2.^46^^,^^47^ Fundamental to many of these processes are inflammation and activation of immune responses, which in severe COVID-19 may be associated with cytokine storm.^48^

## Inflammatory Mechanisms of COVID-19, and Cardiovascular Consequences

Acute and chronic inflammatory responses are at the core of COVID-19 pathology,^49^ as clearly evidenced by the fact that dexamethasone is the only effective therapy in hospitalized patients, as demonstrated by the **R**andomised **E**valuation of **COV**ID-19 Th**er**ap**y** (RECOVERY) trial.^50^^,^^51^ Severe immune dysregulation is characteristic of COVID-19 and ranges from peripheral blood lymphopenia to splenic atrophy, as reported in postmortem examinations.^49^ Circulating levels of proinflammatory cytokines are elevated in patients with COVID-19 and include classical cytokines, such as interleukin (IL)-6 and tumour necrosis factor (TNF)-a, as well as IL-7, IL-2, granulocyte macrophage colony-stimulating factor, and C-X-C motif chemokine 10 *(*CXCL10*),* components of the cytokine release storm.^51^^,^^52^ Increased IL-6 is a clinical biomarker for cardiovascular morbidity and a predictor of mortality in COVID-19.^51^^,^^53^ Inflammation mediates cardiovascular pathology, acting directly on cardiac and vascular cells, and through further propagation of cardiovascular inflammation.

Mechanistically, COVID-19–related cytokines, such as IL-6, IL-17, and TNF-a, induce oxidative stress and inflammation in endothelial and vascular smooth muscle cells, promoting micro- and macro-vascular disease.^54^^,^^55^ Oxidative stress is also key in IL-6–induced increases of adhesion molecule expression in endothelial cells. In addition to causing inflammation, a rapid surge in cytokine production is cardiotoxic, inducing conduction abnormalities, atrial fibrillation, cardiac fibrosis, and heart failure, phenomena observed in the acute phase of severe COVID-19.^56^ Whether the cytokine storm and the IL-6 increase in COVID-19 are transient or sustained processes remains unclear, but monitoring these biomarkers may be important, as they may be predictive of complications in long-term COVID-19.

Although initial studies focused on cytokines that are involved in the cytokine storm, subsequent analysis identified several distinct cellular immunophenotypes in patients with COVID-19. These studies identified inflammatory cells that may be responsible for rapid overproduction of cytokines in COVID-19. Using single-cell RNA sequencing (scRNA-seq), a new immune phenotype in COVID-19 has been described, including a heterogeneous interferon-stimulated gene signature, downregulation of HLA class II, and a developing neutrophil population.^57^ These features are related to severe outcomes, and therefore also to cardiovascular pathologies,^58^^,^^59^ Although peripheral lymphopenia is a feature of severe COVID-19, high-dimensional cytometry revealed activation of T-cell and B-cell subsets in a proportion of patients with plasmablast responses reaching > 30% of circulating B cells.^59^^,^^60^ Interestingly, another immunophenotype was seen in patients who presented with lymphocyte activation comparable to that in healthy controls. In COVID-19, cluster of differentiation (CD)8 T-cell subset skewing and activation patterns were observed, which can be linked to T cell–derived cytokines^60^ observed during cytokine storm. Such hyperactivated T lymphocytes are characterized by large proportions of C-C motif chemokine receptor 6 (CCR6)+ T helper 17 cells CD4+ cells as well as human leukocyte antigen–DR isotope (HLA-DR)+ and CD38+ CD8+/CD4+ T cells, as well as the presence of cytotoxic granules in cytotoxic T (CD8) cells. Hyperactivated T cells and monocytes may account for, at least in part, the severe immune injury observed in cardiovascular disease.^56^^,^^61^^,^^62^

### COVID-19 and cardiovascular inflammation

Cardiac injury and acute myocarditis are recognised complications of acute viral conditions in general.^63^ Myocyte necrosis and mononuclear cell infiltrates are reported in cardiac biopsies from COVID-19 subjects. The most common pathologic cause of myocyte necrosis appears to be microthrombi.^64^ This finding is consistent with numerous early reports of fulminant myocarditis in COVID-19.^65^ However, the actual extent of myocarditis in COVID-19 is difficult to establish.

**Figure 3:**
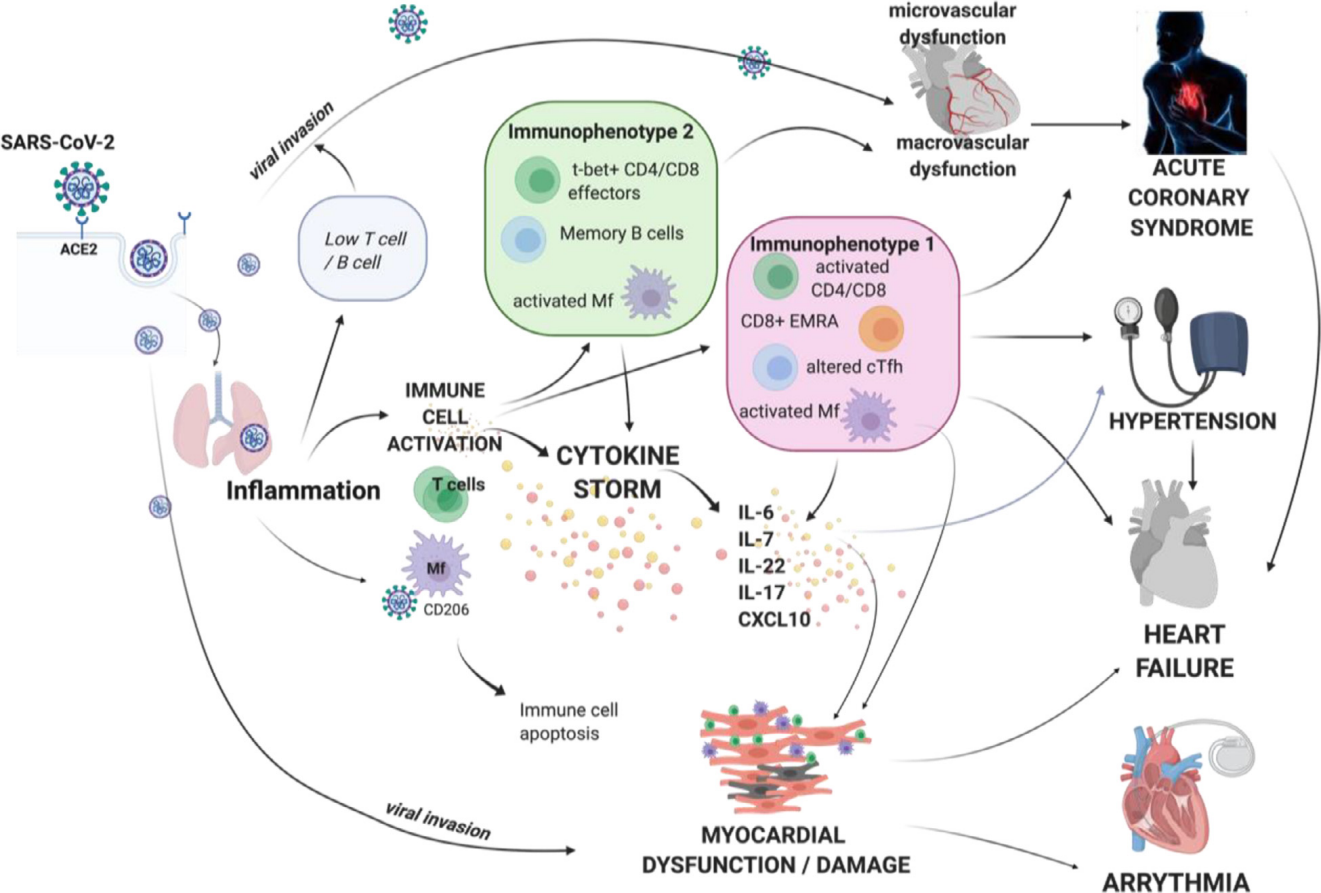
Central role of inflammation and cytokine storm in mediating cardiovascular consequences of COVID-19. Severe acute respiratory syndrome coronavirus 2 (SARS-CoV-2) infection affects the cardiovascular system in 2 ways—through direct viral invasion using entry receptor angiotensin-converting enzyme (ACE)2, and by evoking severe immune response resulting in cytokine storm characterized by systemic overproduction of cytokines such as interleukin (IL)-6, IL-7, IL-22, IL-17, and C-X-C motif chemokine ligand 10 (CXCL10). These cytokines further perpetuate inflammation and cause activation and dysfunction of various cell types in the heart, vasculature, and brain, leading to cardiovascular manifestations. Immunophenotyping studies show that cellular responses in COVID-19 differ among patients and may take the form of several immunophenotypes. CD, cluster of differentiation; cTfh, T follicular helper cells; EMRA, effector memory expressing CD45RA; Mf, macrophage; t-bet, t-box transcription factor.

SARS-CoV2 may directly infect human cardiac myocytes, as demonstrated using inducible pluripotent stem cells-derived cardiac myocytes and human myocardial slices, leading to viral replication inside cardiac myocytes.^66^ SARS-CoV-2–induced cytotoxic and proapoptotic effects lead to cardiomyocyte dysfunction. SARS-CoV-2 infection of cardiomyocytes *in vitro* was inhibited by the antiviral drug remdesivir.^66^ Viral infection and myocarditis in COVID-19 may also exacerbate features of cytokine storm and contribute to further heart muscle dysfunction.^67^ Cardiac injury in models of viral myocarditis leads to innate immunity activation with macrophage infiltration and overproduction of proinflammatory cytokines,^68^ influencing acquired immune responses involving CD4+ T helper cells and cytotoxic CD8+ T cells. ACE2 and other putative SARS Co-V2 receptors are found on numerous cell types in the heart, such as pericytes, and their expression is increased in cardiovascular disease including heart failure.^69^ Accordingly, myocardial dysfunction may be caused by severe systemic inflammation and cytokine storm as well as by direct infection of cardiac myocytes. Together, these effects drive a vicious circle of cardiac inflammation and dysfunction in COVID-19, mediating both short- and long-term cardiovascular consequences of this disease (Fig. 3).

Inflammation and immune responses have also been demonstrated in the vascular system, and there is emerging evidence that many of the cardiovascular complications and systemic pathologies associated with COVID-19 are due to endotheliitis and vasculitis.^70^, ^71^, ^72^, ^73^ Moreover, long-term cardiovascular consequences of COVID-19 may be associated with sustained cytokine increase and persistent cardiovascular inflammation. In particular, cellular immune changes related to distinct immune phenotypes of activated (CD14+CD16+) monocytes as well as lymphocytes have been implicated in long-term cardiovascular sequelae of COVID-19.^74^

### Immunomodulation in COVID-19

Considering the importance of the immune system and inflammation in COVID-19, there has been enormous interest in targeting these processes therapeutically. Trials are currently under way to address the efficacy of immune-targeted therapies in the prevention of severe COVID-19, which will have clear implications for its cardiovascular comorbidities.This approach includes therapeutic targeting of the IL-6 receptor (IL-6R) with tocilizumab, which has been used in preventing and treating cytokine release storm in cancer, treatment of arthritis, and giant cell or Takayasu arteritis and colchicine, a nonspecific anti-inflammatory drug.^75^^,^^76^ Therefore, targeting of the cytokine release storm in COVID-19 may be an attractive possibility, and it would also have protective cardiovascular effects.

## Hypertension

Initial studies at the onset of the pandemic reported a high prevalence of hypertension and other comorbidities and mortality from COVID-19.^77^^,^^78^ In a case-series study of 5700 patients with COVID-19 in New York City, hypertension (56.6%), obesity (41.7%), and diabetes (33.8%) were the most frequent comorbidities.^77^ In a large study of over 72,000 patients with COVID-19 (confirmed, suspected, or clinically diagnosed), the overall case fatality rate was 2.3%, but this increased significantly in the presence of comorbidities (10.5% for cardiovascular disease, 7.3% for diabetes, and 6% for hypertension).^79^ Findings from many studies in various countries show that patients with COVID-19 and hypertension have a higher mortality risk, compared with those without hypertension.^77^, ^78^, ^79^

The relationship between hypertension and COVID-19 is complex, and some studies have not reported an association. In patients with laboratory-confirmed COVID-19 admitted to intensive-care units at 65 US hospitals, there was no impact of hypertension on COVID-19 outcomes, although a body mass index > 40 and coronary artery disease were independent predictors of 28-day mortality.^80^ A multivariable analysis conducted on adults with confirmed COVID‐19 hospitalized at 2 hospitals in Wuhan, China reported that hypertension marginally increased the risk of severe infection and increased risk of mortality only in combination with diabetes or other comorbidities.^81^ The OpenSAFELY analysis^82^ incorporating primary-care data from 17,278,392 patients in England showed that when adjusted only for age and sex, hypertension was associated with a significantly increased risk of death in patients with COVID-19. The authors also found strong evidence of interaction with age, with hypertension associated with a higher risk of death up to the age of 70 years, and a lower risk at ages above 70 years.^82^ High blood pressure is a predictor of heart failure during hospitalization, and increased variability of systolic blood pressure and diastolic blood pressure is associated with increased risk of mortality and intensive-care unit admission.^83^^,^^84^ Increase in fatal and adverse respiratory outcomes have also been demonstrated across the spectrum of blood pressure categories—normotensive through grade I, grade II, and grade III hypertension.^85^

### Indirect effect of COVID-19 on hypertension

Diagnosis, monitoring, and management of hypertension are likely to have been adversely affected by the COVID-19 pandemic, with a reduction in primary care visits^86^—in particular face-to-face appointments—fewer clinic blood pressure measurements, and a lack of public health screening events. Patients with hypertension may also have been less inclined to seek out healthcare visits, owing to unwillingness to burden services, to shielding because of high clinical risk, or to fear of contracting the SARS-CoV-2 virus, leading to late presentation of cardiovascular disease, and poorer outcomes.^87^

The COVID-19 pandemic has highlighted socioeconomic and health inequalities in many countries worldwide. There is an established link between socioeconomic factors (such as education level, income level, and occupation) and the risk of hypertension and rate of blood pressure control.^88^ It is estimated that, compared to those from the least-deprived areas, those from the most-deprived areas are more likely to have hypertension and cardiovascular complications, with some ethnic groups being disproportionately affected—greater rates in Blacks and Asians compared to Whites.^89^ This increased risk may beexplained, in part, by lifestyle factors, including obesity, diet, physical inactivity, and alcohol intake.^90^ COVID-19 has adversely affected those of lower socioeconomic status, for whom mortality rates are twice as high as those in the least-deprived areas.^91^^,^^92^

### Long-term effect of COVID-19 on hypertension

Long COVID is a distinct condition, post–COVID-19, of unknown cause, but it is likely due, at least partly, to an inflammatory reaction and vasculitis.^70^^,^^93^ Even patients without symptoms of thromboembolic disease after COVID-19 may show signs of organ damage.^94^ The presence of considerable ongoing cardiovascular inflammation after recovery from COVID-19 illness is important because it may herald a considerable burden of hypertension and target organ damage down the line.^95^^,^^96^ The combination of potential residual heart and vascular inflammation, along with perturbation of the RAS post–COVID-19 diagnosis, may represent a nidus for new-onset hypertension and heart failure and an insidious feature of long-COVID. The burden of hypertension (from both new-onset and prevalent) as a consequence of the COVID-19 pandemic is unknown, but given the scale of the infection, especially among the young, this impact is a major concern for the future.

### RAS inhibitors, hypertension, and COVID-19

Considering the role of the RAS, and specifically ACE2 in COVID-19 infection and pathophysiology, there has been enormous debate as to whether antihypertensive drugs that inhibit the RAS impact disease severity.^97^, ^98^, ^99^, ^100^, ^101^ Angiotensin-converting enzyme inhibitors (ACEIs) and angiotensin-receptor blockers (ARBs) are considered first-line antihypertensive agents, but they also play a crucial role in the management of patients with renal disease, heart failure, myocardial infarction, and other cardiovascular disorders. Both ACEIs and ARBs reduce the AT_1_R-mediated effects of Ang II and decrease activity of the traditional ACE/Ang II/AT_1_R axis, with possible unmasking of the ACE2/Ang (1-9) and Ang (1-7) pathway.

These effects on the protective arm of the RAS, together with reduced AT_1_R activity, are thought to provide further tissue protection and to constitute an important part of the multifaceted mechanism of action of RAS blocking agents. It is therefore conceivable that patients at highest risk of more severe forms of COVID-19 benefit from the tissue protection offered by these drugs. Apart from immediate effects of ACEIs and ARBs on vascular tone and blood pressure, there are numerous beneficial long-term effects related to their antifibrotic, antiproteinuric, and anti-inflammatory actions. However, with ACE2 being the main receptor for the SARS-CoV-2 spike protein, there has been some concern that upregulation of the ACE2 axis could increase the receptor availability for SARS-CoV-2 host cell entry and infection and more-severe COVID-19.

These concerns are based primarily on experimental data in rodent models. For example, studies in hypertensive rats treated with the ACEI lisinopril or the ARB losartan caused upregulation of cardiac ACE2 mRNA expression.^102^ It was concluded that increased Ang II metabolism by ACE2 contributes to the antihypertensive effects of these drugs.^102^ This upregulation of ACE2 mRNA caused concerns in the initial stages of the COVID-19 pandemic. Driven more by theoretical considerations than by conclusive data, a number of hypothesis and opinion papers were published, causing confusion in the field as to whether ACEIs and ARBs should be withdrawn in COVID-19 patients.^103^

On the other hand, some studies reported that inhibitors of the RAS have beneficial effects in patients with severe COVID-19.^104^^,^^105^ There is now a large body of evidence that confirms that inhibitors of the RAS do not affect the risk of COVID-19. A large study in the Lombardy region of Italy showed that, because of the higher prevalence of cardiovascular risk factors, patients with COVID-19 were more likely to take RAS blocking agents but that the use of these drugs was not independently associated with the risk of COVID-19.^106^ Another study in New York City hospitals found no association between the likelihood of a positive COVID-19 test and use of any of the 5 major classes of antihypertensive agents.^107^ Other studies in the UK and China reported that patients on ACEIs or ARBs were at reduced risk of severe COVID-19 and that these drugs were not associated with increased risks of receiving care in an intensive-care unit.^108^^,^^109^ Meta-analyses of studies into disease severity and mortality have further confirmed that ACEIs and ARBs are not associated with either all-cause mortality or severe COVID-19 disease.^109^ Overall, there is now extensive and unequivocal evidence that ACEI s and ARBs can and should be continued in patients with COVID-19. Based on these data, learned societies, guideline committees, and other scholars have published statements that withholding RAS blocking agents places patients at risk of immediate and long-term sequelae of hypertension and other cardiovascular diseases that outbalances the theoretical risk related to increased expression of a SARS-CoV-2 receptor.^110^, ^111^, ^112^

In clinical practice, however, there are situations in which RAS blocking agents may need to be withheld, as for example, in patients with acute kidney injury or hyperkalemia, and particularly in patients with severe COVID-19 who are hemodynamically compromised. Likewise, it should be noted that there is no evidence that introduction of RAS blocking agents in patients with COVID-19 who have not been on such treatment prior to contracting the disease is associated with any better or worse outcome. Decisions to withhold ACEIs or ARBs should always be based on the clinical situation—independent of SARS-CoV-2 infection. Use of these drugs should always be guided by their evidence-based indications in cardiovascular and renal protection, and not by possible effects on the course of COVID-19. Once COVID-19 has been successfully treated, patients should undergo thorough cardiovascular risk assessment, and RAS blocking agents as well as other primary and secondary preventative measures should be started or reintroduced based on a patient's risk profile. The evolving evidence that SARS-CoV-2 infection increases cardiovascular risk in its own right should also be taken into account.

## Thromboembolic Disease

COVID-19, especially in hospitalized patients, is associated with significant arterial and venous thrombotic complications, including myocardial infarction, ischemic stroke, pulmonary embolism, and venous thromboembolism.^113^, ^114^, ^115^ Early and consistent clinical observations in China and New York indicated that biochemical indices of coagulation were abnormal in COVID-19, with almost 100% of patients with severe disease having mild thrombocytopenia and elevated levels of D-dimer.^116^^,^^117^ Since then, other prothrombotic abnormalities have been described in COVID-19, including increased levels of fibrinogen degradation products (FDPs), factor VIII, and antiphospholipid antibodies and decreased levels of protein C, protein S, and antithrombin.^118^ Elevated levels of D-dimer and FDPs are closely associated with increased severity of disease and mortality, with D-dimer being an independent risk factor for death.^119^^,^^120^ High levels of D-dimer are common in severe illness and are associated with seriousness and death in many severe viral infections, including Ebola, influenza, human immunodeficiency virus, and Dengue.^121^^,^^122^ However, recent studies indicate that arterial and venous thrombosis risk is higher in patients with COVID-19 than that of other forms of viral pneumonia, suggesting that pathophysiological processes are independent of immobilization associated with hospitalization.^123^

Initial studies described the coagulopathy of COVID-19 as DIC. However DIC severity correlates with platelet number and prolonged prothrombin time, and not with fibrinogen and FDPs, which is what is observed in COVID-19. Accordingly, the coagulopathy of COVID-19, typically defined by hypercoagulation associated with elevations in D-dimer and FDPs with mild thrombocytopenia and prolonged prothrombin time, may be distinct from DIC and likely reflects dysregulated hemostasis.^124^

### Histopathologic evidence that COVID-19 is associated with microvascular thromboembolic disease

The first autopsy series from patients with COVID-19 in Wuhan described features of acute respiratory distress syndrome (ARDS) and evidence of small-vessel occlusion, highlighting associated pulmonary microvascular thromboembolic disease.^125^ More recent postmortem studies have provided further pathologic details, including bilateral acute changes with diffuse alveolar damage with vascular congestion, intra-alveolar edema, hemorrhage, proteinaceous exudate, macrophages, denudation and reactive hyperplasia of pneumocytes, and patchy inflammatory cellular infiltration comprising multinucleated giant cells, lymphocytes, (CD4+ve), eosinophils, and neutrophils. A consistently reported feature in postmortem analysis from many case series is microvascular thrombi, neutrophil extracellular traps (networks of extracellular neutrophil-derived DNA), and neutrophil–platelet aggregates contributing to extensive microvascular damage and thrombotic occlusion.^126^^,^^127^ These vascular changes have been attributable, in part, to dysregulation of the endothelial ACE2 receptor with associated bradykinin-mediated lung edema and prothrombotic state.^127^ Beyond the lungs, autopsy findings reveal widespread microthrombi in many organs, including the heart, kidneys, and lungs, and to a lesser extent, the brain.^128^ These phenomena may underlie multisystem organ failure in patients with severe forms of COVID-19, especially in African Americans.^129^

### Mechanisms of COVID-19–associated coagulopathy

The pathophysiology of thromboembolism in COVID-19 vs non–COVID-19 disorders seems to be more platelet-sensitive, with viral-mediated endothelial inflammation, and hypercoagulability associated with increased concentrations of coagulation factors, acquired antiphospholipid antibodies, and decreased concentrations of endogenous anticoagulant proteins. Factors that likely contribute to thromboembolic disease in COVID-19 include a combination of immobility, systemic inflammation, platelet activation, endothelial dysfunction, and stasis of blood flow, which promote coagulation and consequent microvascular and macrovascular thrombosis.^130^ Whether these processes are specific to SARS-CoV-2 infection or rather are a thromboinflammatory consequence of severe viral disease is still unclear.

*In vitro* studies demonstrated that SARS-CoV-2 infection promotes activation of platelets, neutrophils, and endothelial cells, with associated activation of coagulation factors, thrombin generation, fibrin production, increased plasminogen activator inhibitor-1 (PAI-1):tissue plasminogen activator (t-PA) ratio, and production of proinflammatory cytokines, processes that promote hypercoagulation.^131^ In particular, direct viral infection of pneumocytes and endothelial cells promotes an immune and inflammatory response characterized by activation of T cells, neutrophils, macrophages, monocytes, and platelets, leading to cytokine production (IL-1, IL-6, IL-10, TNF), increased PAI-1 expression, and consequent thrombus formation.^114^ Microthrombi in COVID-19 typically contain fibrin, platelets, neutrophils, and neutrophil extracellular traps (NETs), which are tangles of DNA from degenerated neutrophils.^129^ NETs further contribute to hypercoagulation by stimulating the extrinsic pathway and activating platelets.^132^

### Thromboprophylaxis in COVID-19

Although there is a clear association between hypercoagulable states and COVID-19, to what extent SARS-CoV-2 increases the risk of thromboembolic disease remains unclear. Some studies failed to show differences in hospital-associated venous thromboembolism in patients with COVID-19 vs patients with non–COVID-19 illness, suggesting that the coagulopathy is not specific to the virus, but rather is due to the overall illness severity and complications of the disease.^133^ Nevertheless, clinical guidelines suggest that thromboprophylaxis should be considered for all hospitalized patients with COVID-19 in the absence of contraindications.^113^^,^^128^^,^^134^

Early recognition and management of thromboembolism risk, based on C-reactive protein or D-dimer levels, and impending cytokine storm, based on serum ferritin, was associated with improved COVID-19 survival and hospital outcomes in a traffic light–driven personalized care approach.^135^ Current guidelines from the American College of Chest Physicians (ACCP) suggest use of prophylaxis with low-molecular weight heparin or fondaparinux rather than direct oral anticoagulants or fractionated heparin in hospitalized patients with COVID-19 who do not have contraindications, such as bleeding.^136^ However, optimal anticoagulation strategies are still unclear, and prospective clinical trials to determine the best therapeutic approaches are awaited.

## Heart Disease

Infection with viral pathogens has been suggested to associate with an increased risk of myocardial infarction (MI) and cardiovascular risk, from the early 20th century, with a study reporting the highest incidence of heart disease within the first 7 days of infection.^136^ Conversely, during the initial global spread of COVID-19, a reduction in the reported incidence of acute MI was observed, compared with that in previous years.^137^^,^^138^ This reduction was mainly due to behavioural changes by patients. They were more likely to die at home by delaying medical contacts, or to eventually present ‘late.’^139^ These behaviours can be explained by social anxiety relating to hospitals, social distancing measures, and reduction in usual outpatient activities may be implicated.^138^ Presenting late, with more complex illness, inevitably will be more likely to increase persisting chronic sequelae.

### Ischemia and non-ischemic myocardial injury

Systemic pathogenic infection may cause predisposition to acute type 1 MI due to increased levels of circulating inflammatory cytokines in COVID-19 and resultant macrophage activity within atherosclerotic plaques, leading to coronary plaque rupture and thrombosis. Acute SARS-CoV-2 infection is also associated with a prothrombotic and pro-coagulable state; when combined with risk factors such as diabetes, which is associated with impaired fibrinolysis and increased platelet activation, coronary thrombosis may occur.^140^

Type 2 MI secondary to supply and demand mismatch may occur during increased cardiac requirement in the presence of flow-limiting obstructive coronary disease, reduced blood oxygen concentration from COVID-19–related respiratory failure, arrhythmia, shock syndromes, and acid–base or electrolyte disequilibrium. Although cardiac enzymes are elevated in about 40% of patients hospitalized with COVID-19, type 2 MI should be diagnosed only in the presence of symptoms of myocardial ischemia, new ischemic electrocardiogram changes, the development of pathologic q-waves, or evidence of new regional wall motion abnormalities or loss of viable myocardium on imaging.

### Myocardial disease and myocarditis

Cardiovascular injury is associated with history of prior cardiovascular disease, and elevated cardiac enzymes in the context of COVID-19 are associated with poorer outcomes compared with other causes of non-acute coronary syndromes or myocardial injury.^140^ Direct effect on the heart secondary to viral infiltration has been reported but appears to be uncommon.^141^ Cardiac pericytes and cardiomyocytes express ACE2 transmembrane receptor proteins, and fusion with the S protein of SARS-CoV-2 coronavirus is proposed as a source of cell invasion.^142^ However, to-date reports of direct cardiac infiltration in deceased patients at autopsy describe evidence of virus particles within the interstitium or macrophages, rather than within cardiomyocytes.^143^

Pathology studies have identified cardiac lymphocytic or eosinophilic infiltration either at autopsy or following endomyocardial biopsy in patients with COVID-19. The majority of reports are case studies or series with a small sample size, which limits assessment of incidence of myocarditis in hospitalized patients with COVID-19.^144^ Rather than being a direct effect of viral invasion, myocarditis in patients with COVID-19 has been reported to be secondary to cytokine-induced inflammatory myocarditis.^144^

Severe alterations to systemic microvascular and endothelial function have been reported in patients with COVID-19, particularly in those requiring mechanical ventilation.^145^ Coronary microvascular and endothelial dysfunction are therefore potential mechanisms of myocardial damage and persistent symptomatology following infection. In addition, there are reports of diffuse systemic vasculitis, and endothelial involvement may also contribute to impaired microvascular function in patients with COVID-19.^70^, ^71^, ^72^^,^^146^ Microvascular thrombosis has previously been demonstrated at autopsy within pulmonary tissue, and this may also be a cause of coronary microvascular necrosis or dysfunction.^147^ The results of mechanistic imaging studies that feature assessment of coronary microvascular perfusion and myographic vascular function following SARS-CoV-2 infection in patients with concomitant coronary assessment are awaited.^148^

Direct myocardial injury, inflammation, and stress may contribute to Takotsubo cardiomyopathy, a well documented acute cardiotoxic complication of COVID-19 infection.^149^^,^^150^ Mechanisms implicated in COVID-19–related cardiomyopathy include catecholamine surge and cytokine storm.

### Arrhythmia and sudden cardiac death

Atrial fibrillation is the most frequently encountered arrhythmia newly diagnosed in patients with COVID-19, occurring in approximately 1 in 5 hospitalized patients and associated with increased likelihood of mortality.^151^ Premature ventricular complexes, ventricular tachycardia, and bradycardia have also been reported, although it is noted that preexisting rhythm abnormalities were present in a proportion of these patients.^152^ Sudden cardiac death has been reported in case studies; however, QTc prolonging medications were prescribed in these patients, including quinolone antibiotics, or hydroxychloroquine, which has been shown to have no benefit in hospitalized patients.^153^

### Heart failure

COVID-19 infection may predispose patients to developing acute heart failure either because of unmasking of subclinical preexisting heart failure or as a result of direct cardiac involvement. Mechanisms underlying SARS-CoV-2–induced heart failure include virus-induced infiltration of inflammatory cells, proinflammatory cytokines, endothelial injury, micro-thrombosis, and hypoxia secondary to respiratory failure.^154^ Hospitalized COVID-19 patients have a high likelihood of heart failure with preserved ejection fraction (HFpEF) that is associated with cardiac structural and functional abnormalities and myocardial injury and potential long-term heart disease.^155^ Accordingly, detailed screening and cardiac assessments, including echocardiographic determination of left ventricular diastolic function and cardiac biomarkers, have been suggested in the routine care of COVID-19 patients.^156^

Heart failure patients on advanced therapies, including those needing heart transplantation, require special care and involvement of advanced heart failure team members because they are at very high risk due to immunosuppression and hemodynamic instability.^156^ The International Society for Heart and Lung Transplantation guidelines suggest withholding immunosuppressive drugs in moderate-to-severe presentations of COVID-19.^157^ Successful heart transplantation has been described for COVID-19–associated post-infectious fulminant myocarditis.^158^

## Diabetes and COVID-19

In addition to hypertension and obesity, diabetes is strongly associated with COVID-19.^159^^,^^160^ Large population studies reported that people with type 2 diabetes are more than twice as likely to have died from COVID-19 than those without diabetes in the background population (after adjustment for age, sex, ethnicity, social deprivation, and geographical region).^161^ The risk is even higher in people with type 1 diabetes.^159^^,^^160^ Patients with diabetes who developed COVID-19 that was either fatal or required care in the intensive-care unit have more comorbidities and complications (eg, retinopathy) but also poorer glycemic control and higher rates of previous ketoacidosis or hypoglycemia hospitalization.^162^

The mechanisms by which diabetes status, and in particular high blood glucose, predisposes patients to poorer outcomes are currently under intense investigation. It is highly relevant in this regard that the ACE2 receptor is expressed on pancreatic β-cells, potentially predisposing patients to cell damage and loss of endogenous insulin secretion.^163^ Clinical experience indicates that people with diabetes who develop COVID-19 are more likely to develop acute metabolic decompensation, including ketoacidosis, which is itself associated with poorer outcomes. In keeping with the hypothesis of direct toxicity of SARS-CoV-2 to β-cells, increased rates of diagnosis of type 1 diabetes have been reported.^163^^,^^164^

However, additional mechanisms are clearly in play. Hyperglycemia is associated with elevated levels of proinflammatory cytokines, in particular IL-6: the resultant proinflammatory milieu is associated with susceptibility to infection with coronaviruses.^163^ Moreover, associated oxidative stress facilitates entry of the virus into host cells and activation of hypoxia-inducible factor-1α, promoting rapid viral replication and the development of cytokine “storm”—a syndrome that often signals the sharp deterioration of people with COVID-19 after a few days of illness.^163^^,^^164^ Such pathways are now being further investigated in more detail in large cohorts, particularly the **P**ost-**hosp**italization **COVID**-19 Study (PHOSP-COVID), which is currently recruiting 10,000 hospitalized survivors of COVID-19 in the UK.^165^ For example, it is not yet clear to what extent the benefits of therapeutic administration of dexamethasone in oxygen-requiring patients with diabetes and COVID-19 are offset by exacerbation of hyperglycemia.^166^

## The Kidney, Cardiovascular Disease, and COVID-19

Although renal involvement was not a major feature of the early reports of COVID-19 from Wuhan, it is now clear that together with the vascular system and heart, the kidneys are often affected in COVID-19 infection severe enough to require hospitalization. The clinical manifestations of renal involvement in COVID-19 can vary in severity from hematuria and/or proteinuria, to acute kidney injury (AKI) and the need for renal replacement therapy (RRT;, i.e., dialysis or hemofiltration).^167^, ^168^, ^169^ In patients with severe illness requiring management in intensive-care units, the proportion of patients requiring RRT is generally reported to be 20%-30%.^170^^,^^171^ AKI in patients with COVID-19 may lead to volume overload that could exacerbate preexisting chronic heart failure, leading to poor outcomes.

The major risk factors associated with developing biochemical AKI and the need for RRT in COVID-19 are generally similar to those associated with more severe COVID-19 infection; these include male gender, diabetes, non-White race, obesity, preexisting chronic kidney disease, hypertension, and age.^170^, ^171^, ^172^ Increasing COVID-19 disease severity is also associated with increasing risk of requiring RRT. Although any critical illness, such as pneumonia, major surgery, trauma, and sepsis, is associated with a risk of AKI and subsequent need for RRT, emerging data suggest that for an equivalent disease severity, COVID-19 infection appears more likely to provoke AKI.^173^ Similarly, in comparison to seasonal influenza, COVID-19 has a dramatically elevated risk of AKI and need for RRT.^174^

Numerous pathophysiological mechanisms have been proposed to explain why the preponderance of severe COVID-19 infections cause AKI. First, as with any critical illness, shock and renal hypoperfusion will lead to renal ischemia and acute tubular necrosis. The role of systemic inflammation and cardiovascular disease in the setting of COVID-19 may well potentiate AKI, although the specific contribution is unknown. As in cardiac cells, the virus has been demonstrated to directly infect and replicate in kidney cells.^175^ In the setting of severe COVID-19 immune dysregulation, complement types of dysregulation have all been postulated as being implicated in the development of kidney disease.^176^ Furthermore, hypercoagulability and thromboembolic disease associated with COVID-19 may further compromise renal perfusion, and this may lead to transient renal ischemia, or at its most extreme, renal infarction has been described.^177^

The predominant message from epidemiologic studies of COVID-19 is that severe COVID-19 is associated with a prevalence and severity of AKI out of keeping with the severity of renal insult observed with similar critical illnesses. Whether there is a specific effect of SARS-CoV-2 on the kidney is slightly less clear. Much of the original rationale for the disproportionate effect of SARS-CoV-2 on the kidney rested on the high expression of ACE2 in the proximal tubule of the kidney, suggesting the viral invasion may be specific to the kidney, occuring in a manner similar to the invasion of lung tissue.^178^ Some postmortem studies report that SARS-CoV-2 exhibits renal tropism with evidence of direct infection with viral glomerular predilection.^179^ However, other renal histology of postmortem studies of patients with severe COVID-19 has mainly demonstrated severe acute tubular necrosis, which may be a nonspecific finding that simply represents severe critical illness with limited or no evidence of viral infection in the kidney.^180^

Percutaneous renal biopsy is infrequently performed in patients with severe COVID-19 and may therefore be reserved for patients with atypical features, such as disproportionate severity of AKI compared to the clinical course of the illness or heavy proteinuria. In those series of patients with COVID-19 who have clinically indicated kidney biopsies, a range of renal pathologic lesions have been observed, supporting the notion that COVID-19 nonspecifically triggers renal lesions, including acute tubular necrosis, thrombotic microangiopathy, collapsing glomerulopathy, and various glomerulonephritides.^181^^,^^182^ Notably, SARS-CoV-2 was not demonstrated in renal tissue in these native and kidney biopsy transplant series, making it hard to draw conclusions about the specific nature of the renal insult directly attributable to COVID-19.

The presence of AKI is associated with increased risk of mortality in patients with COVID-19, although whether this simply represents a marker of severe infection or is a specific implication of AKI is challenging to determine.^182^^,^^183^ Knowledge of the longer-term implications of cardiovascular and renal involvement in COVID-19 is still evolving. It seems likely that many patients with kidney disease will not return to their pre-COVID-19 renal function. Longer-term follow-up studies will inform this issue. Existing data suggest that 25%-35% patients have not returned to baseline kidney function at the time of hospital discharge and hence have *de novo or* more severe CKD than they did before COVID-19 infection.^168^^,^^169^^,^^183^

## Postural Orthostatic Tachycardia Syndrome and COVID-19

Early in the pandemic, it became apparent that symptoms of COVID-19 could persist after the acute illness, and that many patients who recover from COVID-19 infection experience symptoms for many months after recovery. This condition, called ”long COVID-19.” or ”post-acute sequelae of SARS-CoV-2 infection” is associated with multiple cardiopulmonary and neurologic symptoms, including severe chronic fatigue, palpitations, chest pain, breathlessness, and dysautonomia, features characteristic of postural tachycardia syndrome (POTS).^184^, ^185^, ^186^ POTS impacts heart rate, blood pressure, and cardiac function and can be caused by many factors, including viral infections.^187^ Although it remains unclear whether SARS-CoV-2 triggers POTS, emerging evidence indicates a close association between COVID-19 and POTS-like symptoms. Current treatment strategies focus primarily on lifestyle modifications, and salt and fluid repletion.^185^, ^186^, ^187^ The potential health burden of long COVID-POTS is significant, which has prompted a statement paper on the topic by the American Autonomic Society.^188^

## Management of Cardiovascular Disease in Patients at Risk of COVID-19

It is beyond the scope of the present review to discuss specific protocols in the treatment of patients with cardiovascular disease who are at risk of COVID-19, but the reader is referred to current guidance and opinion papers.^189^, ^190^, ^191^, ^192^, ^193^ In general, management decisions in the treatment of COVID-19 patients with preexisting cardiovascular disease should be considered on a case-to-case basis. Cardiovascular patients should be protected as much as possible from exposure to SARS-CoV-2–infected individuals. Unless otherwise contraindicated, vaccination against SARS-CoV-2 should be encouraged in cardiovascular patients .

## Conclusion

Although there is a clear association between cardiovascular disease and COVID-19, it should be highlighted that many of the studies are retrospective and hence subject to bias and confounding. Distinguishing the very strong effect of age on COVID-19 outcomes from that of other comorbidities whose prevalence increase with age, namely hypertension, diabetes, and other cardiovascular diseases, is challenging. However, resolving this relationship is critical, as this will have implications for management of patients. In the same light, although heart injury seems to be common in patients with severe COVID-19, the long-term health implications and potential lingering effects of cardiovascular damage remain unclear. Moreover, the relevance of cardiovascular disease in SARS-CoV-2–positive, asymptomatic COVID-19 patients is unknown, and as stated by Anthony Fauci, MD, director of the National Institute of Allergy and Infectious Diseases and reported by Abbasi,^194^ the cardiac effects ”may be clinically inconsequential, or could lead to chronic effects.” In addition, the long-term impact of the indirect effects of COVID-19, such as delayed treatment of cardiovascular disease, is still unclear.

There is still no curative therapy for COVID-19, but the successful repurposing of drugs such as remdesivir and dexamethasone, together with successful immunization programs will likely improve the situation. However, as variants of SARS-CoV-2 emerge for which current vaccines offer reduced protection, it is increasingly likely that the pandemic will continue in some form for several years, with cardiovascular complications of COVID-19 remaining a clinical challenge. This prospect has led to urgent calls for increased research into relevant mechanisms and improved prevention and care of patients with cardiovascular and cardiometabolic disease.
